# Cry3Bb1-Resistant Western Corn Rootworm, *Diabrotica virgifera virgifera* (LeConte) Does Not Exhibit Cross-Resistance to DvSnf7 dsRNA

**DOI:** 10.1371/journal.pone.0169175

**Published:** 2017-01-06

**Authors:** William Moar, Chitvan Khajuria, Michael Pleau, Oliver Ilagan, Mao Chen, Changjian Jiang, Paula Price, Brian McNulty, Thomas Clark, Graham Head

**Affiliations:** Monsanto Co., St. Louis, Missouri, United States of America; Institute of Plant Physiology and Ecology Shanghai Institutes for Biological Sciences, CHINA

## Abstract

**Background and Methodology:**

There is a continuing need to express new insect control compounds in transgenic maize against western corn rootworm, *Diabrotica virgifera virgifera* (LeConte) (WCR). In this study three experiments were conducted to determine cross-resistance between the new insecticidal DvSnf7 dsRNA, and *Bacillus thuringiensis* (*Bt*) Cry3Bb1; used to control WCR since 2003, with field-evolved resistance being reported. Laboratory susceptible and Cry3Bb1-resistant WCR were evaluated against DvSnf7 dsRNA in larval diet-incorporation bioassays. Additionally, the susceptibility of seven field and one field-derived WCR populations to DvSnf7 (and Cry3Bb1) was assessed in larval diet-overlay bioassays. Finally, beetle emergence of laboratory susceptible and Cry3Bb1-resistant WCR was evaluated with maize plants in the greenhouse expressing Cry3Bb1, Cry34Ab1/Cry35Ab1, or DvSnf7 dsRNA singly, or in combination.

**Principal Findings and Conclusions:**

The Cry3Bb1-resistant colony had slight but significantly (2.7-fold; P<0.05) decreased susceptibility to DvSnf7 compared to the susceptible colony, but when repeated using a field-derived WCR population selected for reduced Cry3Bb1 susceptibility, there was no significant difference (P<0.05) in DvSnf7 susceptibility compared to that same susceptible population. Additionally, this 2.7-fold difference in susceptibility falls within the range of DvSnf7 susceptibility among the seven field populations tested. Additionally, there was no correlation between susceptibility to DvSnf7 and Cry3Bb1 for all populations evaluated. In greenhouse studies, there were no significant differences (P<0.05) between beetle emergence of susceptible and Cry3Bb1-resistant colonies on DvSnf7 and Cry34Ab1/Cry35Ab1, and between DvSnf7 and MON 87411 (DvSnf7 + Cry3Bb1) for the Cry3Bb1-resistant colony. These results demonstrate no cross-resistance between DvSnf7 and Cry3Bb1 against WCR. Therefore, pyramiding DvSnf7 with Bt proteins such as Cry3Bb1 and Cry34Ab1/Cry35Ab1 will provide a valuable IRM tool against WCR that will increase the durability of these Bt proteins. These results also illustrate the importance of using appropriate bioassay methods when characterizing field-evolved resistant WCR populations.

## Introduction

Corn rootworm-protected *Bacillus thuringiensis* (*Bt*) maize has been rapidly adopted on farms across the mid-western U.S. Corn Belt, where corn rootworm (CRW) (*Diabrotica spp*.) have historically caused devastating damage and yield loss to maize, *Zea mays* L., estimated at approximately $1 billion [1–2]. *Bt* Cry proteins used for CRW control in Corn Belt maize fields include: VT Triple PRO^®^ (Cry3Bb1; available since 2003); Herculex^®^ RW (Cry34Ab1/Cry35Ab1; available since 2005); Agrisure^™^ CB/RW (mCry3A; available since 2007), and Duracade^™^ (mCry3A and e.Cry3.1Ab; available since 2014) [3–7]. SmartStax^®^ maize (MON 89034 × TC1507 × MON 88017 × DAS-59122-7), commercially available since 2009, was the first product to offer CRW protection via a pyramid of two distinct *Bt* protein mechanisms of action, Cry3Bb1 and the Cry34Ab1/Cry35Ab1 binary toxin, and has been commercialized using either a 5% structured refuge or a 5% seed blend refuge in the Corn Belt. Pyramiding traits in this way has the potential to significantly increase the time until resistance develops [8].

There is a continuing need to find and express new insect control compounds in transgenic maize against western corn rootworm, *D*. *virgifera virgifera* (LeConte) (WCR), at least partly due to WCR resistance development to *Bt* maize. Although there currently are five *Bt* proteins (three single and one binary toxin) expressed in commercial transgenic maize to control WCR, resistance in at least some field populations to Cry3Bb1 and Cry34Ab1/Cry35Ab1, and partial-full cross-resistance between Cry3Bb1, mCry3A and eCry3A.1Ab have been reported [9–14]. Therefore, there currently are no traits in commercially available transgenic maize plants for WCR control for which no field-evolved, or at least partial cross-resistance has been reported.

Monsanto Company has developed biotechnology-derived maize, MON 87411, that expresses a double-stranded RNA (dsRNA) and the Cry3Bb1 Bt protein to control WCR. For the dsRNA component, MON 87411 contains a suppression cassette that expresses an inverted repeat sequence designed to match the *Snf*7 gene sequence of WCR. The *D*. *virgifera* Snf7 (DvSnf7) protein is a class E vacuolar sorting protein and belongs to the ESCRT (Endosomal Sorting Complex Required for Transport)-III complex, which has been shown to be essential for sorting of transmembrane proteins [15–16]. The expression of the suppression cassette results in the formation of a dsRNA transcript containing a 240 bp fragment of the WCR *Snf7* gene (DvSnf7). Upon consumption, the plant-produced dsRNA in MON 87411 is recognized by the CRW’s RNA interference (RNAi) machinery resulting in down-regulation of the targeted DvSnf7 gene leading to malfunctioning of the deubiquitination of proteins and autophagy in both midgut and fat body tissues, leading to WCR death [17]. MON 87411 also contains a *cry3Bb1* gene that produces a modified *B*. *thuringiensis* (subsp. *kumamotoensis*) Cry3Bb1 protein to protect against CRW larval feeding. For the next generation of CRW control, Monsanto Company and Dow AgroSciences have used molecular and conventional breeding techniques to develop the combined trait corn product MON 89034 × TC1507 × MON 87411 × DAS-59122-7 (SmartStax^®^ PRO) that confers insect resistance against both key lepidopteran pests and CRW, as well as tolerance to the herbicides glyphosate and glufosinate ammonium. This product, therefore, will contain three Bt proteins; Cry3Bb1 and the Cry34Ab1/Cry35Ab1 binary toxin (all proteins found in SmartStax^®^) as well as the DvSnf7 dsRNA to control CRW.

To determine the potential durability of DvSnf7 expressed as a single trait, or pyramided with Bt proteins, the potential for cross-resistance between DvSnf7 and the Bt proteins currently in the marketplace must be assessed. One of the most widely used methods to determine cross-resistance between insecticidal compounds expressed in plants (to date, essentially all compounds have been Bt) is *in vitro* assessment of binding competition. Bt proteins typically bind to specific midgut receptors, and alterations in receptor binding is the most widely reported mechanism of field-evolved Bt resistance in insects, with most alterations occurring in cadherins, aminopeptidase N’s (APN’s), and alkaline phosphatases (ALP’s) [18]. Insecticidal dsRNA has not been shown to bind to specific midgut receptors; Cappelle *et al*. [19] reported the involvement of clathrin-mediated endocytosis and two Sid-1-like transmembrane proteins in double-stranded RNA uptake in the Colorado potato beetle (*Leptinotarsa decemlineata*) midgut. Additionally, Li *et al*. [20] reported reduced susceptibility (refractoriness) to dsRNA in the laboratory-selected dipteran, *Bactrocera dorsalis* due to down regulation of *chc*, *cogt3*, *light*, and other genes in the endocytic pathway involved with dsRNA uptake. Other potential RNAi-resistance mechanisms include up-regulation of nucleases and defects in dsRNA processing or systemic spread [21–22]. Although single nucleotide polymorphisms (SNP’s) in the target sequence also have been suggested, studies evaluating the host spectrum of DvSnf7 based on sequence homology and 21 bp matches concluded that DvSnf7 resistance due to SNP’s is highly unlikely [23–24]. To date, no evidence exists indicating overlap between the mechanisms of action of *Bt* and dsRNA, and, therefore, no cross resistance between *Bt* Cry proteins (including Cry3Bb1) and DvSnf7 dsRNA is expected. Levine *et al*. [25] conducted bioassays with Cry3Bb1 and DvSnf7 to determine possible joint effects when combined. Using the surrogate southern corn rootworm (SCR), *D*. *undecimpunctata howardi*, they observed no interactions between Cry3Bb1 and DvSnf7, further demonstrating that Cry3Bb1 and DvSnf7 have different mechanisms of action, implying differences in resistance mechanisms. This is one reason why stacking dsRNA with *Bt* proteins has been recommended to increase trait durability [21,26]. However, as there currently are no reported shared mechanisms of resistance between Bt and dsRNA, *in vitro* methods to evaluate cross resistance are not available.

The other widely used method to evaluate cross resistance between two insecticidal compounds is the use of an insect population that is resistant to one of the target insecticidal compounds. The advantages of using such a population (especially a field-evolved resistant population) are that cross resistance may be directly measured and the mechanism of resistance need not be known. The purpose of this study was to determine if cross-resistance exists between Cry3Bb1 and DvSnf7 dsRNA in WCR using both diet (larval) and plant (adult emergence) bioassays with a field-evolved Cry3Bb1-resistant WCR colony.

## Materials and Methods

### Test and Control Substances

All test and control substances for diet bioassays were produced by Monsanto Co. (St. Louis, MO). DvSnf7 968-mer (2.210 mg/ml; Lot # 11331164) was used in diet-incorporation bioassays. The DvSnf7 968-mer is the predominant RNA transcript produced by the DvSnf7 suppression cassette in maize, and in addition to the 240bp insecticidal dsRNA, contains portions of the CaMV 35S promoter, maize *hsp70* intron, and pea *E9* 3’ untranslated region from PV-ZMIR10871 [16,27]. The insecticidal DvSnf7 240-mer (27.75 mg/ml; Lot # 131004-BR221) was used in diet-overlay bioassays. Nuclease free water (UltraPure Invitrogen, Carlsbad, CA) was used as the buffer in both diet bioassays. Cry3Bb1 (Lot # 11309151, 9.9 mg/ml) was used to test for Cry3Bb1 resistance in putative Cry3Bb1-resistant WCR in diet bioassays. Cry3Bb1 storage buffer consisted of 10 mM Sodium Carbonate/Bicarbonate, 0.1mM EDTA, pH 10.0.

Materials used in plant bioassays are listed in Table 1. To determine the contribution of the individual insecticidal compounds in MON 87411 (a maize event expressing Cry3Bb1 and DvSnf7) against WCR to overall MON 87411 toxicity, plants were produced expressing either Cry3Bb1 or DvSnf7 alone that represented expression levels in the commercial event containing both insecticidal compounds. To ensure the expression of Cry3Bb1 and DvSnf7 was similar to that expressed in MON 87411, Cry3Bb1 or DvSnf7 were expressed in the same construct as in MON 87411 except that either *cry3Bb1* or *DvSnf7* was replaced with a marker gene that expresses β -glucuronidase (GUS), the most frequently used marker gene in plants [28]. Therefore, for *DvSnf7*/*GUS*, *Cry3Bb1* present in MON 87411 was replaced with *GUS* leaving only *DvSnf7*. In the case of *Cry3Bb1*/*GUS*, *DvSnf7* present in MON 87411 was replaced with *GUS* leaving only *Cry3Bb1*. Expression levels of DvSnf7 expressed in *DvSnf7*/*GUS* were determined to be similar to DvSnf7 expression in MON 87411 and expression levels of Cry3Bb1 expressed in *Cry3Bb1*/*GUS* were determined to be similar to Cry3Bb1 expression in MON 87411 and MON 88017 (Monsanto, unpublished data).

**Table 1:** Transgenic Corn Rootworm-active Compounds Evaluated Against Susceptible and Cry3Bb1-resistant Western Corn Rootworm.

Treatment	Protein/dsRNA
Non-Traited	None
MON 88017	Cry3Bb1
DAS-59122-7	Cry34Ab1/Cry35Ab1
MON 87411	Cry3Bb1 + DvSnf7 dsRNA
DvSnf7/GUS	DvSnf7 dsRNA at expression levels similar to MON 87411
Cry3Bb1/GUS	Cry3Bb1 at expression levels similar to MON 87411
MON 87411 × DAS-59122-7	Cry3Bb1 + DvSnf7 dsRNA and Cry34Ab1/Cry35Ab1

### Western Corn Rootworm (WCR) Populations

Three WCR colonies were evaluated in diet-incorporation bioassays. The Cry3Bb1-susceptible (Gass-S) non-diapausing colony was obtained by Monsanto from the USDA laboratory (Brookings, SD) in November, 2012. Since that time, this colony has been reared by Monsanto on non-traited maize. The Waterman (WMND) colony is a susceptible non-diapausing colony developed and continuously reared on non-traited maize at Monsanto that also was originally obtained from the USDA laboratory (Brookings, SD). Gass-S and WMND colonies have been determined to be susceptible to Cry3Bb1 and MON 88017 (Cry3Bb1-expressing) maize plants [Monsanto unpublished data, 12]. The Cry3Bb1-resistant (Gass-R) non-diapausing colony was created from a 2010 field-collected population from Iowa from a field planted in Cry3Bb1 maize for seven consecutive years that showed reduced susceptibility to Cry3Bb1. It was back-crossed with the Gass-S non-diapausing WCR strain three times and selected for Cry3Bb1 resistance three times using MON 88017 maize plants [9]. The Gass-R colony was obtained by Monsanto from Aaron Gassmann (Iowa State University) in March, 2012 and has been continuously reared on MON 88017 maize since. Prior to the initiation of DvSnf7 diet bioassays, Cry3Bb1 diet overlay bioassays of the Gass-R and WMND colonies were conducted (using methodologies similar to those described for DvSnf7 diet overlay bioassays below) to document the level of Cry3Bb1 resistance in Gass-R. Gass-R showed no differences in mortality from the untreated control at 588.2 μg Cry3Bb1/cm^2^, whereas there was > 50% mortality at 11.1 μg Cry3Bb1/cm^2^, and 100% mortality at 88.2 μg Cry3Bb1/cm^2^ for the WMND colony. Therefore, Gass-R conservatively has at least 50-fold resistance to Cry3Bb1 compared to WMND, though resistance is likely greater than 100-fold.

Diapausing WCR populations used in diet-overlay bioassays were field-collected from various locations throughout the Corn Belt in 2012 (Table 2). Populations were collected from product performance inquiry (PPI) fields as part of the conditions of registration for corn rootworm-protected products, and had been previously evaluated for Cry3Bb1 susceptibility by Custom Bio-Products (Maxwell, IA). The laboratory Cry3Bb1-susceptible non-diapausing WCR eggs were obtained from Crop Characteristics (Farmington, IL). This colony was established about 20 years ago from wild WCR populations collected in MN. Additionally, a field-derived WCR population with reduced Cry3Bb1 susceptibility (MW13) was also evaluated. MW13 was created by crossing 12 PPI and seven non-PPI (NPPI; WCR populations collected from fields where no changes in product performance was reported) field populations with the WMND colony and selecting twice on MON 88017 maize plants prior to bioassays. Prior to the initiation of DvSnf7 diet-overlay bioassays, Cry3Bb1 diet overlay bioassays of the MW13 and WMND colonies were conducted (using methodologies similar to those described for DvSnf7 diet overlay bioassays below) to document the level of Cry3Bb1 susceptibility in MW13. MW13 exhibited less than 50% mortality at the highest concentration tested (500 μg Cry3Bb1/cm^2^), whereas the LC_50_ value (± 95% FL) for WMND was 22.58 μg Cry3Bb1/cm^2^ (3.27–70.98). Therefore, MW13 conservatively has at least 22-fold reduced susceptibility to Cry3Bb1 compared to WMND.

**Table 2:** Western Corn Rootworm Populations Collected from 2012 Product Performance Inquiry (PPI) Fields^1^ and tested against Cry3Bb1 or DvSnf7.

ID	Location
111564221^1^	Cedar, NE
111568810^1^	Lincoln, NE
111569293^1^	Lac qui Parle, MN
111569492_2^1^	Bureau, IL
111571869C^1^	Kit Carson, CO
120038737^1^	Mercer, IL
120024975^1^	Knox, IL
Routine Monitoring^2^	IA, IL, MN, NE
WMND^3^	Waterman, IL
MW13^4^	Waterman, IL
Diet 12CONTROL01^5^	Crop Characteristics
Diet 12CONTROL02^5^	Crop Characteristics

^1^ Populations collected from Product Performance Inquiry (PPI) fields.

^2^ Populations collected from non-traited fields in IA, IL, MN and NE for routine monitoring.

^3^ Lab non-diapause susceptible population from Monsanto insectary, Waterman, IL.

^4^ Field-derived lab population created by combining 12 PPI and 7 NPPI populations and crossing with WMND and selected on MON 88017 for two generations.

^5^ Lab non-diapause susceptible population from Crop Characteristics, Farmington, MN.

Two WCR colonies (Gass-S and Gass-R) were evaluated in adult emergence plant bioassays. The Gass-R non-diapausing colony was obtained from Aaron Gassmann as described previously and was reared on MON 88017 for five generations before plant bioassays were initiated. Prior to initiation of plant bioassays, Cry3Bb1 diet incorporation bioassays were conducted (using diet incorporation methodologies similar to those described below) to document Cry3Bb1 resistance in Gass-R. Gass-R showed no differences in mortality from the untreated control at 3 mg Cry3Bb1/ml diet, whereas Gass-S showed 71% mortality at 3 mg Cry3Bb1/ml diet, confirming that Gass-R was resistant to Cry3Bb1.

### Bioassays

For diet-incorporation bioassays, the Gass-R and Gass-S colonies could not be bioassayed concurrently for all replicates due to limited egg availability and asynchrony of the colonies. Therefore, the WMND laboratory susceptible colony was included as a control in all replicates for both the Gass-R and Gass-S colonies. Eggs for all colonies were held in soil at 10°C to limit development until incubation at 25°C, 70% relative humidity (RH) and 0:24 L:D photoperiod for 13 d. Prior to hatch, WCR eggs and soil were placed in a 60 mesh screen with a 30 mesh screen on top. Lukewarm (about 22°C) water was used to remove soil from eggs. Eggs were then rinsed in a 150 ml beaker with DI water. Eggs were allowed to settle and floating eggs (likely non-viable), debris, and water were poured off. Eggs were sterilized as described by Pleau *et al*. [29] and monitored for larval hatch. WCR larvae (<24 h old) were exposed to DvSnf7 (968-mer) in diet-incorporation bioassays. Six DvSnf7 concentrations plus a buffer-only control were used. WCR diet was prepared using autoclaved Millipore water, Agar (Serva #11393), Bio-Serv Southern corn rootworm diet (F9757B), and other propriety ingredients. Two-hundred microliters of the diet plus DvSnf7 mixture was dispensed into each well of a 96-well plate and allowed to cool under a laminar flow hood [Labconco (Cat. # 36100410784), Kansas City, MO]. Prior to infesting, an anti-microbial solution was overlaid onto the diet and allowed to dry under a laminar flow hood. Once dry, each well was infested with a single WCR larva that was ≤ 24 hours old. Plates were sealed with mylar film using a hot tacking iron and one hole per well was punched with a #000 insect pin for ventilation. Plates were labeled with the treatment, colony ID, and Test-set ID, which included date of infest and replicate. Bioassay plates were stored in a Percival incubator at 27°C, 70% RH, and 0:24 L:D photoperiod for 12 d. After 12 d, the number and instar of the surviving larvae were recorded. Larvae were considered dead if they were immobile. If control mortality exceeded 25%, the replicate was discarded. Diet bioassay replicates were conducted with all colonies using a target number of 32 WCR at each concentration. Bioassays were repeated three times for both Gass-S and Gass-R colonies, and six times for the WMND colony (for the first replicate of WMND, two subsets of 32 insects were included: one subset to compare to Gass-R and one to compare to Gass-S for a total of 64 insects for this replicate).

The susceptibility of WCR field and field-derived populations to DvSnf7 dsRNA was assessed in diet-overlay bioassays. All bioassays were conducted by exposing neonates (<24 h old) to artificial diet, surface-treated with increasing concentrations of the DvSnf7 dsRNA 240-mer. WCR egg dishes were incubated and sterilized as described above. Diet was dispensed into 96-well plates as described above. Twenty microliters of DvSnf7 at varying concentrations were overlaid onto the diet surface and air dried under a laminar flow hood. One WCR neonate larva (< 24 h old) was infested in each well with a fine tipped paintbrush. Plates were sealed with a silicone-adhesive sealing film and ventilated. Bioassay plates were incubated at 27°C, 60% RH for 12 d. At day 12, the number of surviving larvae was recorded. Larvae were considered dead if they were immobile or in first instar. If control mortality exceeded 25%, the replicate was discarded.

For plant bioassays, a single kernel of maize from each treatment (Table 1) was planted into a container and filled with steam-sterilized soil (67% heavy field soil and 33% Metro 200). After planting, containers were maintained in a greenhouse under conditions conducive to growing maize. Each individual container served as a replicate (ten replicates per treatment). When plants reached approximately V2-V3, plant identity was confirmed using either lateral-flow strips (for Cry3Bb1 or Cry34Ab1/Cry35Ab1; Envirologix, Portland, ME) or Seed Quality Technology (SQT) analysis for DvSnf7 dsRNA. For SQT analysis, two leaf samples from each plant were collected and processed per MQC (Molecular Quality Characterization) procedures at Monsanto (St. Louis, MO). For *DvSnf7*, a PCR-based assay designed to detect the 3’UTR was used to confirm the presence of the gene. To ensure there was no contamination in the *DvSnf7* material, several PCR-based assays to detect the gene of interest in other events were also used. Plants that did not test as expected were discarded. When plants reached approximately the V4-V5 leaf stage, all 10 replicates for all treatments for each WCR insect colony were infested with 50 WCR neonates (< 24 h old) [30]. Because egg hatch was not always synchronized, a subset of each treatment was infested on a similar day with ten replicates per treatment reached within a five day period. Plants were infested as follows: Six and eight plants per treatment were infested with Gass-S and Gass-R neonates on October 9, 2013, respectively. Four and two plants per treatment were infested with Gass-S and Gass-R neonates on October 10, 2013, respectively. Approximately 21–28 d after infestation, tassels and dead leaves from maize plants were removed, and each container was placed in a fine beetle-proof mesh net (Pollination tube, Type 2, Vilutis and Co., Frankfort, IL) to monitor beetle emergence. Plants were watered as needed during the beetle emergence period. Each pot was monitored three times per week for teneral beetles. Beetles were collected via aspiration. Containers were monitored until no beetles had emerged for at least 10 d, once emergence had commenced. Beetles were stored in vials containing 70% ethanol until evaluation.

### Statistical Analysis

For diet incorporation bioassays on the three laboratory colonies, probit analysis was conducted to estimate the LC_50_ values along with natural mortality. The SAS procedure PROC NLMIXED was used in the analysis with a randomized complete block design, replicate indicating an assay targeting all three colonies in different dates considered as random [31]. The solution (colony-specific slopes and intercepts) to the model was used to estimate the concentrations required for 50% mortality (LC_50_) which is an estimate after the adjustment for natural mortality. A Goodness of Fit (GOF) test of the probit model (in SAS output; Pearson Chi-Square) was conducted to determine the fit of the concentration-response curves by comparing the expected mortality (including the concentration related mortality as well as the natural mortality) with the raw data. For certain concentrations (0.080, 0.400, and 2.000 μg DvSnf7/gram diet), the predicted numbers of dead or alive were less than five and thus were grouped [32].

For diet overlay bioassays on field and field-derived populations, a separate probit analysis was performed using the SAS procedure PROC PROBIT for each population. The analysis was performed on the pooled data over all plates for each concentration. The natural mortality was a model parameter, and LC_50_ values were estimated as the concentration corresponding to 50% mortality adjusted for natural mortality; when mortality was observed in the zero concentration. For MW13, there was no mortality at the zero concentration, so natural mortality was assumed to be zero, and no adjustment was made.

**Fig 1:**
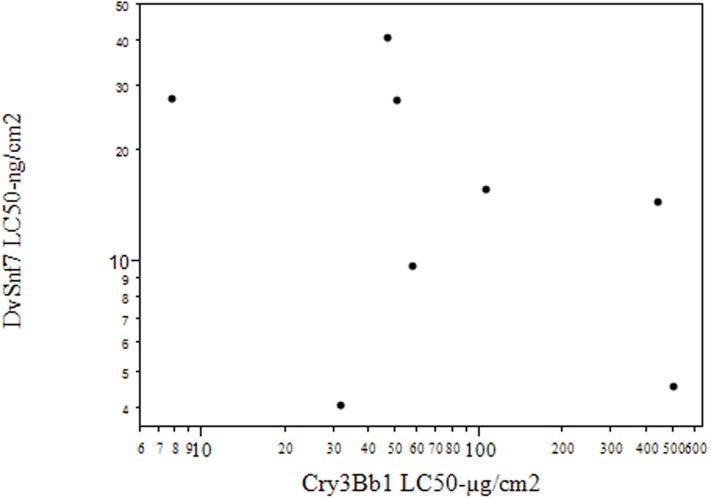
No Correlation (R^2^ = −0.3912) between Cry3Bb1 and DvSnf7 LC_50_ Values in log scale for 2012 Western Corn Rootworm Populations from Product Performance Inquiry (PPI) Fields and a Field-derived Laboratory Population selected for Reduced Cry3Bb1 Susceptibility.

To visualize and analyze any possible correlation in the resistance to Cry3Bb1 and DvSnf7, a linear regression analysis was performed simply by comparing Cry3Bb1 and DvSnf7 LC_50_ values for all field and field-derived populations. Visual inspection of the scatter plot (Fig 1) seems to match well with the analysis result.

For plant bioassays, the percentage of beetle emergence (number of beetles emerging compared to number of larvae infested) was first summarized for each replicate (i.e. plant) and then the means and standard errors over replicates were generated for each treatment and each colony. The emergence percentage in Gass-R and Gass-S colonies was compared in a logistic model with colony as a fixed effect and replicate as a random effect. The SAS procedure PROC GLIMMIX was used for the comparison. However, in treatments MON 87411 and MON 87411 × DAS-59122-7, there was no emergence for colony Gass-S and PROC GLIMMIX could not perform the analysis. Therefore, Fisher’s exact test was used in the comparisons with this colony.

## Results

For diet-incorporation bioassays, the LC_50_ values for DvSnf7 against susceptible WCR were 15 ng/g and 17 ng/g for WMND and Gass-S, respectively (Table 3). Comparisons among the six replicates of the WMND colony showed no significant differences (P = 0.718), indicating assays were consistent over time; no assay related variation needs to be considered when the Gass-R colony is directly compared to the Gass-S colony. The Cry3Bb1-resistant Gass-R colony, with greater than 50-fold resistance to Cry3Bb1, had slight but significantly (2.7-fold) decreased susceptibility to DvSnf7 compared to the susceptible Gass-S colony (Table 3).

**Table 3:** Toxicity of the DvSnf7 968-mer Against Susceptible (Gass-S and WMND) and Cry3Bb1-Resistant (Gass-R) Western Corn Rootworm in Diet-Incorporation Bioassays.

Colony	Total # Insects	LC_50_ Value	95% CL	Slope (Mean±SE)	DF	χ^2^ Value	P Value
Gass-R	614	0.046	0.03–0.07	0.593±0.074	14	20.68	0.110
Gass-S	605	0.017	0.01–0.02	1.944±0.245	5	4.55	0.473
WMND	1452	0.015	0.01–0.02	1/604±0.142	15	11.48	0.718

^1^ μg DvSnf7 968-mer /g diet

For diet overlay bioassays, seven 2012 WCR PPI field, and the MW13 field-derived populations that previously had been tested with Cry3Bb1, were also tested with DvSnf7. All PPI population Cry3Bb1 LC_50_ values were similar to the LC_50_ values for the field populations collected and tested for routine Cry3Bb1 resistance monitoring (IA, IL, MN, NE) with several PPI population LC_50_ values being similar to the two laboratory susceptible colonies (Table 4). In DvSnf7 diet overlay bioassays, there was an approximate 10-fold variation in DvSnf7 LC_50_ values among these same PPI populations (Table 4). Four of the seven PPI populations had significantly (P<0.05) higher DvSnf7 LC_50_ values than the susceptible WMND colony; the LC_50_ value for the WMND colony was similar to the lowest LC_50_ value for the PPI field populations (Lincoln, NE), and about 13-fold lower than the field population with the highest DvSnf7 LC_50_ value (Mercer, IL). Although the field-derived MW13 population had the lowest susceptibility to Cry3Bb1 of any population tested (and at least a 22-fold reduced susceptibility to Cry3Bb1 compared to WMND), the LC_50_ value for MW13 against DvSnf7 was not significantly different (P<0.05) from the DvSnf7 LC_50_ value for WMND. A scatter plot and linear regression analysis showed no significant correlation (R^2^ = −0.3912) between DvSnf7 and Cry3Bb1 susceptibility for the seven PPI field and MW13 populations (Fig 1).

**Table 4:** Toxicity of Cry3Bb1 and the DvSnf7 dsRNA 240-mer against 2012 Western Corn Rootworm Populations from Product Performance Inquiry (PPI) Fields, Routine Monitoring, Field-derived, and Laboratory Susceptible Colonies.

Population Location	Insect #^1^	LC_50_ Values^2^	95% CL	Slope (SE)	P Value^3^
**Cry3Bb1**^4^					
Bureau, IL^5^	360	7.75	2.62–13.30	1.28 (0.26)	0.1303
Cedar, NE^5^	504	57.66	4.95–118.8	1.71 (0.44)	0.0810
Kit Carson, CO^5^	360	50.41	31.81–84.20	0.82 (0.18)	0.5675
Knox, IL^5^	432	105.50	35.06–1523	0.54 (0.21)	0.9551
Lac qui Parle, MN^5^	432	438.99	133.2–5.7 X10^7^	0.63 (0.28)	0.1703
Lincoln, NE^5^	288	31.51	14.10–53.18	1.19 (0.27)	0.1171
Mercer, IL^5^	360	46.86	31.01–65.06	1.59 (0.27)	0.7964
IA, IL, MN, NE (low) ^6^	72	21.17	8.64–33.17	2.60 (0.77)	0.4924
IA, IL, MN, NE (high) ^6^	288	382.38	160.33–1565	0.96 (0.35)	0.5439
MW13^7^	230	>500	-	-	-
Crop Characteristics^8^	360	4.90	0.90–9.86	1.15 (0.27)	0.9596
Crop Characteristics^8^	504	18.62	12.37–24.98	1.51 (0.20)	0.8548
**DvSnf7 dsRNA**					
Bureau, IL	110	27.75	7.45–92.80	0.96 (0.29)	0.5878
Cedar, NE	165	9.70	4.05–17.44	1.36 (0.30)	0.5843
Kit Carson, CO	103	27.38	0.80–72.70	1.51 (0.66)	0.3949
Knox, IL	108	15.72	5.75–32.70	1.40 (0.35)	0.3495
Lac qui Parle, MN	104	14.61	4.78–31.23	1.53 (0.41)	0.6033
Lincoln, NE	137	4.07	0.08–13.91	0.73 (0.25)	0.1249
Mercer, IL	164	40.51	18.35–94.84	1.09 (0.26)	0.1016
MW13	164	4.56	2.34–8.28	1.12 (0.17)	0.6581
WMND^9^	320	2.99	1.50–4.64	1.81 (0.36)	0.8926

^1^ Total number of insects over all replicates (plates) and concentrations

^2^ LC_50_ values: DvSnf7 dsRNA: ng/cm^2^ after 12 days; Cry3Bb1: μg/cm^2^ after 3–4 days.

^3^ P value of the Pearson’s Chi Square test on Lack-Of-Fit

^4^ The F1 generation was tested for all field populations except MW13

^5^ Populations collected from Product Performance Inquiry (PPI) fields.

^6^ Populations collected from non-traited fields in IA, IL, MN and NE for routine monitoring. Out of 11 colonies evaluated for routine monitoring, results include the colony with the lowest LC_50_ value and the colony with the highest LC_50_ value

^7^ Field-derived lab population created by combining 12 PPI and 7 NPPI populations and crossing with WMND and selected on MON 88017 for two generations. The observed mortality is about 22% at the highest concentration tested (500 μg Cry3Bb1/cm^2^), and therefore expectedly LC_50_ > 500 μg Cry3Bb1/cm^2^.

^8^ Laboratory non-diapause susceptible colony from Crop Characteristics.

^9^ Laboratory non-diapause susceptible colony from Monsanto insectary, Waterman, IL

In plant bioassays, the toxicity of the individual insecticidal components in MON 87411 × DAS-59122-7 as well as MON 87411 and MON 87411 × DAS-59122-7, was evaluated against susceptible (Gass-S) and Cry3Bb1-resistant (Gass-R) WCR using beetle-emergence assays in the greenhouse. Beetle emergence of Gass-S for MON 88017, DAS-59122-7 and Cry3Bb1/GUS was reduced and significantly (P<0.05) less than the non-traited control (Table 5). For Gass-R, the number of beetles emerging from the non-traited control was not significantly different (P<0.05) than the Cry3Bb1 entries (MON 88017 and Cry3Bb1/GUS) confirming that the Cry3Bb1-resistant colony was resistant to Cry3Bb1. Beetle emergence of Gass-R and Gass-S from DAS-59122-7 were not significantly different (P<0.05), demonstrating no cross-resistance between Cry3Bb1 and Cry34Ab1/Cry35Ab1. There also were no significant differences (P<0.05) between beetle emergence of Gass-S and Gass-R on DvSnf7/GUS, and between DvSnf7/GUS and MON 87411 for the Cry3Bb1-resistant colony. These results demonstrate that there is no cross-resistance between DvSnf7 and Cry3Bb1 in WCR.

**Table 5:** Mean Percent Beetle Emergence^1^ for Susceptible (Gass-S) and Cry3Bb1-Resistant (Gass-R) Western Corn Rootworm Feeding on *Bt* and RNAi-traited Maize.

Colony	Non-Traited	MON 88017^2^	DAS-59122-7	Cry3Bb1/GUS^2^	DvSnf7/GUS	MON87411^3^	MON 87411 × DAS-59122-7
Gass-S^4^	30.2a	14.4b	11.0b	14.0b	2.6c	0.0d	0.0d
Gass-R^4^	32.0a	40.8a	10.4b	38.2a	4.4bc	3.4c	0.4d

^1^ Mean percent beetle emergence based on 50 insects infested each onto 10 plants

^2^ Mean percent beetle emergence **within a column** was significantly different between the two colonies at the 0.05 level.

^3^ Mean percent beetle emergence **within a column** was significantly different between the two colonies using Fisher’s Exact Test (p <0.05)

^4^ Mean percent beetle emergence with different letters **within a row** are significantly different at the p<0.05 level

## Discussion

With field-evolved resistance in at least some field populations to Cry3Bb1 with partial-full cross resistance to mCry3A and eCry3.1Ab, and field-evolved resistance in at least some field populations to Cry34Ab1/Cry35Ab1, there is a critical need to find and express new insect control compounds in transgenic maize against WCR [9–14]. Monsanto is developing a new WCR product based on dsRNA (DvSnf7), but understanding the potential IRM value of this product requires assessing potential cross-resistance with other WCR traits and proteins. Although *in vitro* studies (typically involving binding parameters) often are used as an indirect method to evaluate potential cross-resistance between Bt proteins, use of a target pest population resistant to one of the Bt proteins is a more direct and preferred method for assessing cross-resistance. In the case of Bt (Cry3Bb1) and dsRNA (DvSnf7), there are no *in vitro* methods to evaluate cross-resistance; the use of a resistant colony such as the Cry3Bb1-resistant WCR colony described in this report is the only viable approach.

Three different experiments were conducted to determine cross-resistance between the new insecticidal compound, DvSnf7 dsRNA, and Cry3Bb1 in WCR. The first experiment compared a laboratory susceptible (Gass-S) and field-evolved Cry3Bb1-resistant (Gass-R) WCR in diet-incorporation bioassays. However, as Gass-S was not synchronous with the Cry3Bb1-resistant colony, another susceptible colony (WMND), in which eggs were readily available, was used in every bioassay to bridge between the two experiments. The LC_50_ values for Gass-S and WMND were 17 and 15 ng/g, respectively, and these values are not significantly different. As both susceptible colonies originated from the non-diapausing WCR strain developed at the USDA-ARS North Central Agricultural Laboratory (Brookings, SD) it is not surprising that the colonies yielded similar results [33]. These values are similar to those reported by Bolognesi *et al*. [16] when corrected for the molecular weight difference between the 968-mer (used in this study) that contains the insecticidal 240-mer and the 240-mer used in Bolognesi *et al*. [16]. Additionally, Levine *et al*. [25] found similar differences between the DvSnf7 968-mer and the 240-mer against SCR reported by Bolognesi *et al*. [16]. This result indicates that the methods used to determine LC_50_ values were robust and consistent with previously published research.

The Cry3Bb1-resistant Gass-R colony, with greater than 50-fold resistance to Cry3Bb1, had slight but significantly (2.7-fold) decreased susceptibility to DvSnf7 compared to the susceptible Gass-S colony. However, this 2.7-fold decreased susceptibility to DvSnf7 between Gass-R and Gass-S falls well within the range of DvSnf7 susceptibility among the seven field populations (in which there was no correlation between DvSnf7 and Cry3Bb1 susceptibility) tested in diet overlay bioassays. When DvSnf7 was evaluated against WMND and the MW13 colony (a field-derived population with at least 22-fold reduced susceptibility to Cry3Bb1 compared to WMND), there was no significant difference (P<0.05) between MW13 and WMND. These results suggest that attributes other than Cry3Bb1 resistance, such as differences in vigor between a recently laboratory-adapted field-evolved Cry3Bb1 resistant colony (Gass-R) and the more inbred laboratory-adapted colonies (Gass-S and WMND), and the inherent variability of conducting WCR bioassays, are more likely the cause for this low but significant level of reduced susceptibility between Gass-R and Gass-S. Such results raise the question of what an appropriate comparator for evaluating resistance and cross-resistance should be [12, 34–35]. In evaluating cross-resistance in field populations between Cry3Bb1, Cry34Ab1/Cry35Ab1, mCry3A and eCry3.1Ab, Zukoff *et al*. [12] recommend using more than one comparator. Recent bioassay results from a diapausing WM WCR colony (only one generation per year and therefore fewer generations of laboratory rearing) also showed a significant decrease in susceptibility to the DvSnf7 968-mer (Monsanto unpublished data) compared to the WMND colony, suggesting that laboratory adaptation of WCR may increase susceptibility to DvSnf7, possibly due to loss of field vigor characteristics.

To better represent the commercial product expressing dsRNA and Bt Cry proteins (MON 87411 × DAS-59122-7), a greenhouse study was conducted comparing laboratory susceptible (Gass-S) and Cry3Bb1-resistant (Gass-R) WCR against single and pyramided Bt and dsRNA-expressing maize plants. To ensure the expression of Cry3Bb1 and DvSnf7 was similar to that expressed in MON 87411, Cry3Bb1 or DvSnf7 were expressed in the same construct as in MON 87411 except that either Cry3Bb1 or DvSnf7 was replaced with a marker gene that expresses β-glucuronidase (GUS), the most frequently used marker gene in plants [28]. In the case of single Cry3Bb1 events, there was no significant difference between MON 88017 and Cry3Bb1/GUS against Gass-S. There was also no significant difference between non-traited, MON 88017 and Cry3Bb1/GUS against Gass-R demonstrating that 1) the Cry3Bb1/GUS treatment accurately represents the expression and toxicity of Cry3Bb1 in MON 87411, and 2) Gass-R was resistant to Cry3Bb1, which is in agreement with the preliminary diet bioassays. For DvSnf7 events, there was no significant difference between DvSnf7/GUS and MON 87411 against Gass-R. These results support the use of GUS as a replacement for an insecticidal compound in a multiple insecticidal gene construct when needing to evaluate single insecticidal compounds in a pyramid. There was no significant difference between Gass-S and Gass-R against Cry34Ab1/Cry35Ab1, further demonstrating no cross-resistance between Cry3Bb1 and Cry34Ab1/Cry35Ab1 as reported previously [9–10]. There was no significant difference between Gass-S and Gass-R against DvSnf7/GUS, and there was no significant difference between DvSnf7/GUS and MON 87411, demonstrating that there is no cross-resistance between Cry3Bb1 and DvSnf7, in agreement with the diet-overlay assay results.

When evaluating the field relevance of differences in susceptibility (possibly due to inherited traits), the longer the bioassay, the more likely the results will be reflective of the field (http://cals.arizona.edu/pubs/crops/az1437/section3.pdf). For evaluating WCR, diet bioassays evaluating larval mortality and development, and plant bioassays evaluating larval mortality and development, and adult emergence, are routinely used [12]. Two advantages of plant bioassays when testing field-evolved resistant WCR are 1) evaluating adult emergence is possible allowing the measurement of the full impact of an insecticidal compound at the end of the insects’ entire life cycle, and 2) potential toxin-plant and WCR-soil interactions can be observed. Therefore, it is not surprising that plant-based results (based on adult emergence) exhibited no significant differences in susceptibility to DvSnf7 between susceptible and Cry3Bb1-resistant WCR compared to the relatively low levels of reduced susceptibility observed in the diet incorporation bioassay results (larval mortality). To date, there are no reports that would suggest that there would be cross-resistance between dsRNA and Bt Cry proteins, and therefore, no cross-resistance between *Bt* Cry proteins (including Cry3Bb1) and dsRNA (including DvSnf7) would be expected [18–20]. This is one reason why stacking dsRNA with Bt proteins has been recommended to increase trait durability [21, 26].

Although this study concentrated on WCR, the primary rootworm pest in the U.S. Corn Belt, expressing a new mechanism of action such as DvSnf7 also will increase the durability of transgenic maize against northern corn rootworm (NCR), *Diabrotica barberi*. Although testing NCR in laboratory or greenhouse bioassays can be challenging, DvSnf7 in MON 87411 has been shown to be highly effective against NCR in field tests (Monsanto unpublished data). Given that the spectrum of toxicity of DvSnf7 is closely related to phylogeny, especially in the coleopteran family Chrysomelidae; Subfamily Galerucinae, that contains the genus *Diabrotica* and the close phylogenetic relationship between WCR and NCR, NCR is expected to be highly susceptible to DvSnf7 with no cross-resistance to Bt proteins as exhibited by WCR [24, 36].

In conclusion, DvSnf7 presents a new mechanism of action against corn rootworm with no cross-resistance to Bt proteins, and will be a valuable addition to Bt maize pyramids such as MON 87411 × DAS-59122-7 in SmartStax^®^ PRO, especially against Cry3Bb1-resistant WCR. Pyramiding DvSnf7 with Cry3Bb1 and Cry34Ab1/Cry35Ab1will provide a valuable IRM tool against WCR that will increase the durability of these Bt proteins.
